# Quantification and reduction of the health sector’s environmental impact

**DOI:** 10.2471/BLT.25.294153

**Published:** 2026-02-03

**Authors:** Rachel Lisa King, Petra Mariaria, Anais Mennecier, Nicolas Nagot, Rosalind Parkes-Ratanshi

**Affiliations:** a Department of Epidemiology and Biostatistics, University of California, San Francisco 550 16th St, San Francisco, CA 94158, United States of America.; b Infectious Diseases Institute, Academy for Innovations and Health, Kampala, Uganda.; c Institut National de la Santé et de la Recherche Médicale (INSERM) Occitanie Méditerranée, Montpellier, France.; d Centre for Public Health, Queens University, Belfast, Northern Ireland.

Climate change is one of the most important threats to planetary and human health.^1^^–^^3^ The carbon footprint of health care, reported as the mass of carbon dioxide (CO_2_) released in the biosphere as a result of the delivery of health-care services, is responsible for 4–5% of greenhouse gas emissions globally.^2^^,^^3^

Health systems and climate change are intertwined because the higher incidence and severity of catastrophic climate events like hurricanes, floods, heat waves, fires and other climate disasters increases the direct and indirect burdens on health systems. As health care advances and becomes more technology-based, the provision of health care is increasingly contributing to climate change through greenhouse gas emissions that harm the environment.^4^ Addressing the environmental impact of health care supports the climate by finding new ways to reduce greenhouse gas emissions and unnecessary waste while improving health outcomes and economic efficiency. The health sector, encompassing research, development, delivery and consumption of services, has a unique obligation to drive climate action while enhancing population, human and wildlife health.^3^ However, existing regulations rarely consider climate risks, which in turn affect human health.

## Ethics and implications 

Responsibility for greenhouse gas emissions overwhelmingly lies with industrialized countries that continue to couple their economic growth to energy demands, including for biomedical research and development. Whereas biomedical products may benefit all people and countries, many of these products are consumed by resource-rich populations, while the health outcomes linked to the climate change caused by these products fall disproportionately on low-resource populations with limited capacity to mitigate these consequences. 

Inequities arise in how interventions are implemented and in earlier decisions, including which research questions are pursued, which diseases receive attention, and how resources are allocated across countries and communities. Actions to mitigate climate change frequently benefit human and wildlife health, often bringing matching, economic gains.^5^ However, benefits and costs are context-dependent and show differences in the outcomes, meaning actions should be driven by and tailored to local environments.^5^

The African continent is one of the most biodiverse on the planet, with multiple different ecosystems and unique plant and animal species.^6^ Although the conservation movement in Africa is growing, with local recognition of the importance of protecting the environment,^7^ African policy-makers and concerned community representatives continue to be essential participants in discussions around measurement of climate emissions as these profoundly affect their human and animal health systems.^6^ If decisions come from Indigenous community members and policy-makers, context-relevant actions may follow.

Clinical trials are a cornerstone of evidence-based medicine. By evaluating the safety, efficacy and potential benefits and risks of medical interventions with scientific rigour, clinical trials provide evidence to advance medical knowledge and human health. Clinical trials today may include an economic evaluation to determine and compare the financial costs of new interventions to inform policy decisions, but evaluation of the environmental costs of health interventions is rare. Yet these costs can directly translate into increased human morbidity and mortality, thus additional human health costs, notwithstanding environment costs.^8^ Adding the costs related to the environment when designing and implementing clinical trials is therefore a logical approach that would enable policy-makers to consider health outcomes alongside economic and environmental costs.

Beyond direct impacts on human health, climate change and health-care-related emissions have extensive consequences on ecosystems. Increased greenhouse gas emissions accelerate land degradation, ocean acidification and biodiversity loss, which affect food security and water quality. Emissions from health-care logistics, pharmaceutical production and waste incineration contribute to air, soil and water contamination.^8^ These environmental degradations feed back into human, wildlife and environmental well-being through reduced air and water quality, as well as fewer ecosystem actions, such as pollination. The inclusion of planetary health indicators within research and policy evaluation would provide a fuller understanding of the trade-offs between human and environmental health, aligning with the One Health and planetary health approaches.

With growing interest in and need to deliver greener health interventions, robust research in this area is a priority.^9^ While a nascent body of literature measuring the climate impact of clinical research exists, trials that compare carbon footprints of the interventions within clinical trials, independent of study-related activities, are scarcer. The relevance of environmental impact assessments within health-care research has been demonstrated in a study, for example, evaluating how infection-control practices can unintentionally increase the carbon footprint of health-care delivery.^10^ This study highlighted that a shift towards single-use disposable laryngoscopes led to higher emissions, resource consumption and waste generation without corresponding infection-control benefits. This work suggested harmonized guidance between infection-control regulations and environmental-sustainability objectives.^10^

Our team is a multidisciplinary collaboration currently measuring the CO_2_ emissions of interventions in two cluster-randomized controlled trials in Uganda and Zambia, where we measure CO_2_ emissions during patient recruitment and at follow-up alongside health and financial outcomes. Our primary objective in the Uganda-based trial is to compare the carbon footprint of anti-retroviral therapy delivery using medical drones versus standard methods (boats) over 24 months on islands of Lake Victoria. In the Zambia-based trial, the objective is to compare the carbon footprint of a post-natal human immunodeficiency virus (HIV) prevention package versus control (national HIV prevention guidelines) study arms also embedded in a cluster-randomized trial in urban and rural Zambia over 12 months. The climate measurement we have adopted is life-cycle assessment. This assessment evaluates the environmental impacts of a product or system throughout its life cycle according to the International Organization for Standardization 14040/14044 standards for environmental management.^11^

## Recommendations

These trials provide a starting point to analyse the combined human health, economic and climate cost questions. Many difficult technical and ethical questions emerge, such as how future interventions can balance the immediate human health gains with non-human and planetary health. 

We propose a structured, integrated conceptual framework that explicitly links health, economic and climate outcomes through implementation science and equity lenses. Our suggested framework recognizes these domains as dynamically interdependent, where health outcomes influence economic costs; economic conditions shape exposure, vulnerability and adaptive capacity to mitigate climate risks; climate events directly and indirectly affect population health and health system performance; and components of the health system directly influence the climate. Implementation science can act as a central conceptual pathway, translating evidence across these domains into real-world policies and service delivery models, while considering context, feasibility and scalability. Equity is embedded as a cross-cutting principle, ensuring that both the design and implementation of interventions explicitly assess differential impacts across populations, settings and structural determinants. This framework could provide a structure for evaluating trade-offs, synergies and consequences of interventions, therefore supporting more sustainable, equitable and policy-relevant decision-making at the health–economy–climate nexus.

One important risk this framework addresses is burden-shifting, that is, when gains in one domain are achieved at the expense of another, such as climate mitigation strategies that increase costs to under-resourced health systems or pass on costs to patients. Without a framework and measurement that identifies and highlights all domains, interventions could shift economic or environmental burdens onto marginalized populations, reinforcing existing inequities. Similarly, greenwashing poses a risk when initiatives are framed as green or climate-responsive without transparently and accurately measuring reductions in emissions, resource use or considering environmental harm across the full implementation pathway. By explicitly linking outcomes across health, economic and climate dimensions, and embedding equity-sensitive indicators, the framework is designed to highlight these risks early, enabling transparent evaluation and course correction before practices are scaled.

When climate outcomes conflict with health or financial outcomes, trade-offs should be evaluated using a transparent, multicriteria approach. Decisions should prioritize safety and quality first, while explicitly and concurrently comparing gains and losses across health, economic and climate domains. Equity considerations are critical, as trade-offs may disproportionately affect specific vulnerable groups, health workers or lower-resource settings. The framework we propose encourages assessment of immediate outcomes and longer-term system resilience and downstream effects. Making these trade-offs explicit supports accountable, contextually appropriate actions.

The challenges scientists in the fields of health and climate face at the intersection of these fields are complex and require fresh, collaborative and innovative thinking. We recommend combining the expertise of clinical researchers, climate and social scientists, health economists, ethicists, policy-makers, community members and communication and sustainability experts. As many team members as possible should be locally based to promote studies that assess contextually relevant social and environmental costing. We must engage communities to promote the inclusion of a climate measurement into clinical and implementation research globally, with input from all the relevant actors that would benefit from, learn and promote their use. By creating a roadmap to identify and measure these impacts on people and the planet, we aim to enhance future design and implementation of trials and interventions that account for the planetary costs and sustainability of interventions. We believe that stimulating new connections between fields and experts in largely disconnected arenas across geographies and cultures to address these crucial questions will reshape how scientists, communities and policy-makers think and act. This reshaping affects decisions on how, where and what to include in the design and implementation of clinical trials and the delivery of health services worldwide.
